# Inhibition of VEGF and Angiopoietin-2 to Reduce Brain Metastases of Breast Cancer Burden

**DOI:** 10.3389/fphar.2017.00193

**Published:** 2017-04-11

**Authors:** Kaci A. Bohn, Chris E. Adkins, Mohamed I. Nounou, Paul R. Lockman

**Affiliations:** ^1^Department of Pharmaceutical Sciences, School of Pharmacy, Texas Tech University Health Sciences Center, AmarilloTX, USA; ^2^Department of Pharmaceutical Sciences, College of Pharmacy, Harding University, SearcyAR, USA; ^3^Department of Pharmaceutical Sciences, School of Pharmacy, West Virginia University Health Sciences Center, MorgantownWV, USA; ^4^Department of Pharmaceutical Sciences, South University, SavannahGA, USA; ^5^Department of Pharmaceutical Sciences, Appalachian College of Pharmacy, OakwoodVA, USA; ^6^Department of Pharmaceutics, Faculty of Pharmacy, Alexandria UniversityAlexandria, Egypt; ^7^Department of Pharmaceutical Sciences, School of Pharmacy, University of Saint Joseph, HartfordCT, USA

**Keywords:** angiogenesis, bevacizumab, brain metastases, permeability, prevention, L1-10

## Abstract

For metastases in the central nervous system, angiogenesis enhances metastatic potential and promotes progression. Primary factors which drive vessel growth are vascular endothelial growth factor (VEGF) and angiopoietin-2. Preclinical models show inhibition of either factor reduces metastases spread and inhibits growth. This work sets out to answer two questions in a preclinical mouse model. First, whether the combined inhibition of VEGF and angiopoietin-2, reduces passive permeability and limits drug uptake into brain metastases; and second, whether this inhibition reduces metastases burden in brain. We observed combinatorial inhibition of VEGF and angiopoietin-2, decreased (*p* < 0.05) angiogenesis and vascular branching in an aortic ring assay and decreased (*p* < 0.05) endothelial wound closure times. Using a brain metastases of breast cancer model (induced by intracardiac injections of brain seeking MDA-MB-231Br cells or 4T1Br cells), we observed, similar to VEGF, angiopoetin-2 expression correlates to increased angiogenesis (*p* < 0.05) and increased lesion permeability. To determine efficacy, animals were administered bevacizumab plus L1-10 (angiopoietin inhibitor) twice per week until neurological symptoms developed. Lesion permeability significantly decreased by ∼50% (*p* < 0.05) compared to untreated lesions, but remained ∼25% greater (*p* < 0.0%) than brain. In subsequent experiments, animals were administered similar regimens but sacrificed on day 32. The number of metastatic lesions developed was significantly (*p* < 0.001) reduced in the bevacizumab group (56%) and combination group (86%). Lesions’ size was reduced in bevacizumab treated lesions (∼67%) and bevacizumab and L1-10 treated lesions (∼78%) developing area < 0.5 mm^2^. In summary, combinatorial inhibition of VEGF and angiopoietin reduces lesion permeability and brain metastatic burden.

## Introduction

Current treatment options of brain and CNS metastases are modest and only expand survival approximately 4 months (Labidi et al., 2009; Pestalozzi, 2009; Goldfarb et al., 2011). Most patients (70%) die within 1 year (Pestalozzi, 2009). Previously, a preclinical model analyzing 2,000 brain metastases of breast cancer found that, while nearly all lesions had increased permeability, only 10% of the lesions were permeable enough to allow drug to accumulate at concentrations which were sufficient to induce cytotoxicity (Lockman et al., 2010). The study suggested that the blood–brain barrier (BBB) and the blood-tumor barrier (BTB) present a significant obstacle in the treatment of brain metastases by limiting drug uptake to subtherapeutic levels (Lockman et al., 2010).

The BBB serves as a functional and structural barrier, limiting passive diffusion of hydrophilic and charged compounds into brain (Abbott et al., 2009). With the occurrence of brain metastases, new vessels are either sprouted from existing BBB vessels or potentially created *de novo* (Groothuis, 2000; Pestalozzi, 2009). BTB vascular structure is distinctive compared to the vascular structure of BBB. BTB has increased permeability, different pattern of transporters regulation, and possibly downgraded blood flow (Hiesiger et al., 1986; Bronger et al., 2005; Gerstner and Fine, 2007).

In metastases, vascular destabilization and accompanying angiogenesis enhance tumor growth (Folkman, 1990), upregulate growth factors such as vascular endothelial growth factor (VEGF) and Ang-2 (Veeravagu et al., 2007), metastatic potential (Fidler and Ellis, 1994; Claffey et al., 1996), and correlate with poor patient outcome (Weidner et al., 1992; Meitar et al., 1996; Uzzan et al., 2004). A primary driver of angiogenesis is VEGF, which is secreted by tumor cells in response to decreased vessel density and hypoxia. VEGF is highly expressed in breast, colorectal, and non-small cell lung carcinoma (Lee et al., 2007; Kadota et al., 2008; Barresi et al., 2010). A secondary driver of angiogenesis is angiopoietin-2 (Ang-2), which activates in response to hypoxia and induces vessel destabilization upon binding the Tie2 receptor (LaManna et al., 2004; Chae et al., 2010). Both VEGF and Ang-2 have been shown to independently induce angiogenesis (Blacher et al., 2001; Teichert-Kuliszewska et al., 2001).

Importantly, VEGF and Ang-2 have been shown to synergistically act to induce endothelial destabilization, increase vascular branching (Chae et al., 2010; Hashizume et al., 2010; Morrissey et al., 2010), and increase angiogenesis (Holash et al., 1999; Zhang et al., 2003). In addition, the two growth factors independently induce formation of endothelial branches in *ex vivo* aortic ring assays, suggesting an angiogenic effect on perivascular cells as well (Nicosia et al., 1997; Iurlaro et al., 2003).

Inhibition of VEGF with bevacizumab, a monoclonal antibody which binds to the VEGF ligand preventing receptor phosphorylation, has been shown to reduce angiogenesis in tumors (reducing vessel branching and growth) (Borgstrom et al., 1996), resulting in slower tumor development (Zhang et al., 2002; Holloway et al., 2006; Roland et al., 2009), reduced metastasis development (Ellis et al., 2000; Verheul et al., 2007), and improved drug delivery through vascular normalization (Tong et al., 2004; Jain, 2005). Several studies evaluating bevacizumab in glioblastoma patients demonstrated that pharmacological treatment reduced brain edema and intracranial pressure, leading to a small increase in progression-free survival (Vredenburgh et al., 2007; Verhoeff et al., 2009). However, a review evaluating bevacizumab in CNS tumors (clinical and preclinical glioma models) showed that bevacizumab decreased the permeability of the contrast agent gadolinium at the BTB (Thompson et al., 2011). Currently, the only bevacizumab data available for brain metastases of breast cancer are two studies which report only the relative risk of hemorrhagic events when taking the drug (Labidi et al., 2009; Besse et al., 2010).

Similarly, the peptide-Fc fusion L1-10 has been shown to block Ang-2 from binding the Tie2 receptor, which also decreases angiogenesis and tumor growth in both prostate cancer (Morrissey et al., 2010) and gliomas (Villeneuve et al., 2008). Additionally, the administration of L1-10 in the presence of different concentrations of cytotoxic drugs increased anti-tumor activity (Brown et al., 2010). Therefore, it has been hypothesized that simultaneous inhibition of VEGF and Ang-2 will decrease tumor growth and/or development (Bergers et al., 2003; Erber et al., 2004; Chae et al., 2010).

In this report, we inhibit both VEGF and angiopoetin-2 in an experimental brain metastases model to determine how this affects metastatic burden in brain. First, we demonstrate that VEGF and Ang-2 act cooperatively to induce angiogenesis *in vitro*; next, we identify the development of hypoxic brain metastases, which exhibit Ang-2 activation. We then evaluated the effects of bevacizumab and L1-10 on metastases formation and permeability of experimental brain metastases. To accomplish this, we injected ∼175 k brain seeking cells (MDA-MB-231-BR or 4T1-BR5 overexpressing Her-2) into the peripheral circulation and allowed experimental brain metastases to develop (Lockman et al., 2010). Ten days after injection of cells, bevacizumab (10 mg/kg) (Lucio-Eterovic et al., 2009) and L1-10 (4 mg/kg) (Villeneuve et al., 2008) were administered in experimental groups. After neurological symptoms developed, we administered ^14^C-AIB or ^14^C-paclitaxel intravenously to determine permeability and drug uptake. We observed that both ^14^C-AIB permeability and ^14^C-paclitaxel drug uptake was significantly reduced. Of importance, we observed a reduction in the number of metastatic lesions in bevacizumab and bevacizumab + L1-10 treated groups.

These data suggest that combinatorial therapy of VEGF blockade and Ang-2 inhibition may be an effective strategy to reduce brain metastases formation. However, this dual target approach also reduced permeability and drug uptake in established brain metastases, which warrants caution in the therapeutic use of VEGF and Ang-2 inhibition.

## Materials and Methods

### Chemicals

^14^C-AIB (specific activity: 55 mC_i_/mmole) was purchased from American Radiolabeled Chemicals (St. Louis, MO, USA). ^14^C-Paclitaxel (specific activity 70 mCi/mmole) was obtained from Moravek Biochemicals and Radiochemicals (Brea, CA, USA). The peptide-Fc fusion, L1-10 was a kind gift from Amgen (Thousand Oaks, CA, USA), and bevacizumab (Avastin^TM^) was purchased from Genentech (San Francisco, CA, USA). Hypoxyprobe^TM^ kit was purchased from HPI Inc. (Burlington, MA, USA). All other chemicals are of analytical grade and were purchased from Sigma (St. Louis, MO, USA).

### Animals

Female NuNu mice (25–30 g) were obtained from Charles River Laboratories (Kingston, NY, USA) and used for the metastasis experiments in this study. Male Fisher 344 rats (300–350 g) also from Charles River Laboratories were used for aortic ring assays. All studies were approved by the Animal Care and Use Committee at Texas Tech University Health Sciences Center, and conducted in accordance with the 1996 NIH Guide for the Care and Use of Laboratory Animals.

### Cell Culture

Human metastatic breast cancer over-expressing Her2 cells (MDA-MB-231-BR-Her2) and 4T1-BR5 murine mammary carcinoma cells were chosen for the experimental brain metastases model. Murine brain endothelial cell cultures (bEND5) were cultured in DMEM medium supplemented with 10% FBS, 1% non-essential amino acids, 1% sodium pyruvate, 1% penicillin/streptomycin/amphotericin B, and 2% 200 mM L-glutamine. All cells were used in passages 1–10 and maintained at 37°C with 5% CO_2._

### Development of Metastases and Administration of Inhibitors

Mice were anesthetized and inoculated with breast cancer cell lines (MDA-MB-231-BR-Her2: 1.75 × 10^5^ and 4T1-BR5: 5 × 10^4^) in the left cardiac ventricle with the use of a stereotaxic device. Tumor cells were confirmed to seed in the brain via an IVIS Lumineer XV *in vivo* animal imager (Caliper Life Sciences, Inc., Part of PerkinElmer, Inc.). Tumors were allowed to develop over ∼4–6 weeks or until neurological symptoms of weight loss, lethargy, or paralysis appeared. As metastases seeded the brain, treatments were administered beginning on day 10 [Bevacizumab (10 mg/kg, i.p., twice weekly) (Lucio-Eterovic et al., 2009) and L1-10 (4 mg/kg diluted in PBS, s.c., twice weekly) (Villeneuve et al., 2008)] and continued until day 32, at which point animals were euthanized as mentioned.

### Injection of Vascular Markers and ^14^C-Paclitaxel

In permeability studies, on day 32, ^14^C-AIB (25 μC_i_/animal; American Radiolabeled Chemicals) was injected intravenously (10-min circulation), animals were sacrificed, brain tissue was removed and placed in isopentane (-65°C). For evaluation of paclitaxel uptake, ^14^C-paclitaxel (25 μC_i_/animal) was injected and allowed to circulate for 120 min, followed by euthanasia and rapid removal of brain tissue for immediate freezing in isopentane. For hypoxia evaluation, animals were injected intravenously with pimonidazole hydrochloride (60 mg/kg, Hypoxyprobe^TM^) and allowed to circulate for 1 min. Afterward, tissues were processed, and immunofluorescence was performed. Brains were sliced at 20 μm thickness using a cryostat (Leica Microsystems, Wetzler, Germany). Direction of brain slice cutting was rostral (anterior) to caudal (posterior). Sections were mounted on glass slides, air dried, and stored at -80°C. The distribution of ^14^C-AIB and ^14^C-paclitaxel in brain and metastases was measured by quantitative autoradiography.

### Immunofluorescence

Tissues were rehydrated briefly in phosphate buffered saline (PBS) and fixed in cold 4% paraformaldehyde (4°C) for 30 min. After three washings of PBS, the slides were then covered in 1% sodium dodecyl sulfate for 5 min and rinsed again with PBS. Slides were submerged in blocking solution of 10% goat serum in PBS for 30 min at room temperature. Primary antibodies diluted in 5% goat serum: CD31 (Clusters of Differentiation 31) (BD Pharmingen, San Diego, CA, USA), Ang-2 (Abcam, San Francisco, CA, USA), Hypoxyprobe^TM^ rabbit antisera (HPI Inc, Burlington, MA, USA) were incubated overnight at 4°C followed by washing. Sections were covered in 0.03% hydrogen peroxide + 0.1% sodium azide for 10 min and subsequently washed. After a second blocking with 10% goat serum for 30 min, slides were incubated with secondary antibodies diluted in 5% goat serum: Alexa Fluor^®^ 594 (red) and Alexa Fluor^®^ 488 (green). DAPI was added to this solution at 1 mg/mL (Invitrogen, 4′,6-diamidino-2-phenylindole, dilactate) and slides were incubated for 1 h at room temperature.

### Migration Assay

Cell migration was evaluated with an *in vitro* wound healing assay. The endothelial cells (bEND5) were cultured in 24 well plates (DMEM supplemented with 10% FBS) to confluence and serum starved for 1 h prior to beginning treatments. After obtaining quiescence, the cultures were wounded with a 0.1 cm^3^ pipette tip in one direction. To remove cellular debris, the wounded cells were washed with PBS. Cells were incubated with DMEM (supplemented with 1% FBS) containing various concentrations of Ang-2 (recombinant human Angiopoietin-2, R&D Systems), VEGF (recombinant human VEGF, R&D Systems), L1-10 (Amgen), and/or bevacizumab (Avastin^TM^ Genentech) at 37°C. Images were taken at initial wound healing, 24, 48, and 72 h, and percentage of wound closure was calculated.

### Rat Aorta Model of Angiogenesis

Male Fisher 344 rats were anesthetized and a mid-line incision was made to reveal the thoracic cavity. The diaphragm was cut and the heart and lungs were removed. The aorta was then excised and placed in 4°C DMEM medium and sectioned into 3 mm segments. Aortic rings were kept on ice until they were embedded into approximately 200 μl of matrigel (BD Biosciences) in 24 well plates. Rings were kept at 37°C for 1 h and 50 μl of matrigel was placed on top of the rings. Rings were allowed to sit for 24 h in DMEM containing 1% FBS at 37°C before treatment was initiated and time point zero photos were taken. Rings were incubated with various concentrations of Ang-2, VEGF, bevacizumab, and/or L1-10 in DMEM containing 1% FBS. Images were taken at 24, 48, and 72 h. Branching morphogenesis was calculated by counting the number of angiogenic branches in three sections (500 μm in size) per ring.

### Assessment of Metastases Development

To evaluate the number and size of metastases developed, ten microscope slides containing five brain slices (20 μm/slice) were randomly selected per animal (*n* = 3–6 animals per treatment group). Metastatic lesions were identified by eGFP fluorescence and then counted in one brain slice per slide, being careful to count only individual tumors rather than large lesions, which might be present in multiple slides. Comprehensive digital microscopy software (3i Slidebook^TM^ 5.0) was used to calculate lesion area.

### Statistical Analysis

Student’s *t*-test was used to determine statistical significance in aortic ring assays, migration assays, and the difference in Ang-2 expression between permeable and non-permeable lesions. Linear regression analysis was used to determine the relationship between ^14^C-AIB permeability independently with Ang-2 expression and metastasis size. One-way ANOVA followed by Bonferroni’s multiple comparisons test was used to evaluate significance of AIB brain space, the number of metastases developed in each treatment group, and the percentage of metastases in each size group. All differences were considered statistically significant at *p* < 0.05. Data are reported as Mean ± Standard Error of Mean (SEM) (GraphPad Prism^®^ version 7.0).

## Results

### *In vitro* Angiogenesis Assays

**FIGURE 1:**
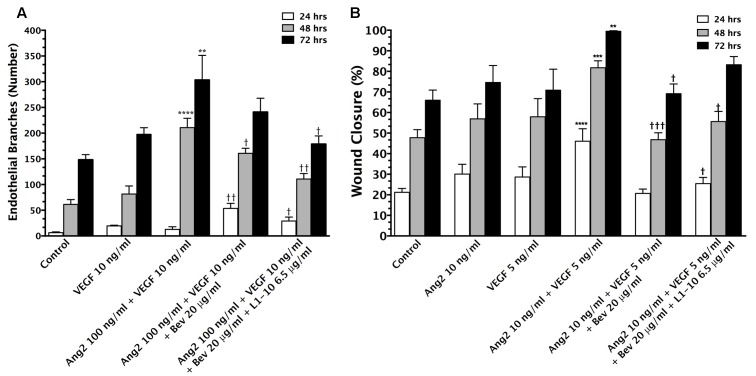
** Vascular endothelial growth factor (VEGF) and Ang-2 promote angiogenesis and can be modulated by inhibition.** The endothelial branching observed from *in vitro* aortic ring angiogenesis assays in the presence of VEGF (10 ng/mL) + Ang-2 (100 ng/mL) and the addition of bevacizumab (20 μg/mL) and L1-10 (6.5 μg/mL) for 24, 48, and 72 h duration of treatment **(A)**. An increase in endothelial branching compared to control is denoted by (^∗^). A reduction in endothelial branching compared to VEGF + Ang-2 is denoted by (^†^). Wound closure of bEND5 cells in the presence of Ang-2 (10 ng/mL) + VEGF (5 ng/mL) and the addition of bevacizumab (20 μg/mL) and L1-10 (6.5 μg/mL) in comparison to Ang-2 (10 ng/mL) and VEGF (5 ng/mL) over 24, 48, and 72 h **(B)**. An increase in percent wound closure relative to control is denoted by (^∗^) and a decrease in wound closure relative to Ang-2 + VEGF is denoted by (^†^). Statistical evaluation was determined by student’s *t*-tests; ^∗^ and ^†^ for *p* < 0.05, ^∗∗^ and ^††^ for *p* < 0.01, ^∗∗∗^ and ^†††^ for *p* < 0.001 and ^∗∗∗∗^ and ^††††^ for *p* < 0.0001.

To confirm that VEGF and Ang-2 synergistically induced angiogenesis *in vitro*, which could be inhibited with bevacizumab and L1-10, we used aortic ring and wound healing assays. Briefly, aortic ring growth (**Figure 1A**) averaged 6.4 ± 1.5, 61 ± 9.4, and 148 ± 10 endothelial branches at 24, 48, and 72 h, respectively. A significant increase (*p* < 0.01) in branching was seen at 48 and 72 h (211 ± 18.2 and 303 ± 47.9 branches) in wells treated with the combination of Ang-2 and VEGF as compared to control. Upon the addition of bevacizumab, branching remained unchanged at 24 h (54 ± 10 branches) but was reduced (*p* < 0.05) at 48 h (161 ± 9.8) and 72 h (241 ± 27 branches). The addition of L1-10 further reduced endothelial branching (*p* < 0.05) at all three time points. Following the aortic ring assays, we completed wound healing assays (**Figure 1B**) to compliment the aortic ring data. Our initial experiments demonstrated that the most effective combinatorial concentration of VEGF and Ang-2 to create wound closure was 10 ng/mL for Ang-2 and 5 ng/mL for VEGF (data not shown). Furthermore, the combinational treatment of VEGF and Ang-2 increased wound closure (*p* < 0.01) at 24 (30% ± 4.8), 48 (57% ± 7.3), and 72 h (76% ± 8.3) compared to control values (21% ± 1.9, 48 ± 3.9, and 66 ± 4.9), Ang-2 values (30.06% ± 4.779, 56.92 ± 7.283, and 74.59 ± 8.283) and VEGF values at (28.66% ± 4.9, 57.92 ± 8.8, and 70.86 ± 10.22) at the same respective times. The addition of bevacizumab resulted in a significant decrease (*p* < 0.05) in noted cell migration. To a lesser extent, this was also seen with the combination of bevacizumab and L1-10. The i*n vitro* studies confirm the angiogenic effects of VEGF and Ang-2 on endothelial cells.

### Metastases Are Hypoxic

In our second set of experiments, we set out to determine if hypoxia was present in the lesions, which would activate Ang-2 expression, increasing angiogenesis. We observed that nearly all lesions have some degree of hypoxia using Hypoxyprobe^TM^ (Pimonidazole HCl) staining; however, regions of hypoxia appeared heterogeneous between all lesions irrespective of metastasis size (data not shown). The variability in tumor vascular density could result in a larger lesion containing less pimonidazole HCl than the smaller lesion (Groothuis, 2000).

### Ang-2 Expression in Metastatic Lesions

**FIGURE 2:**
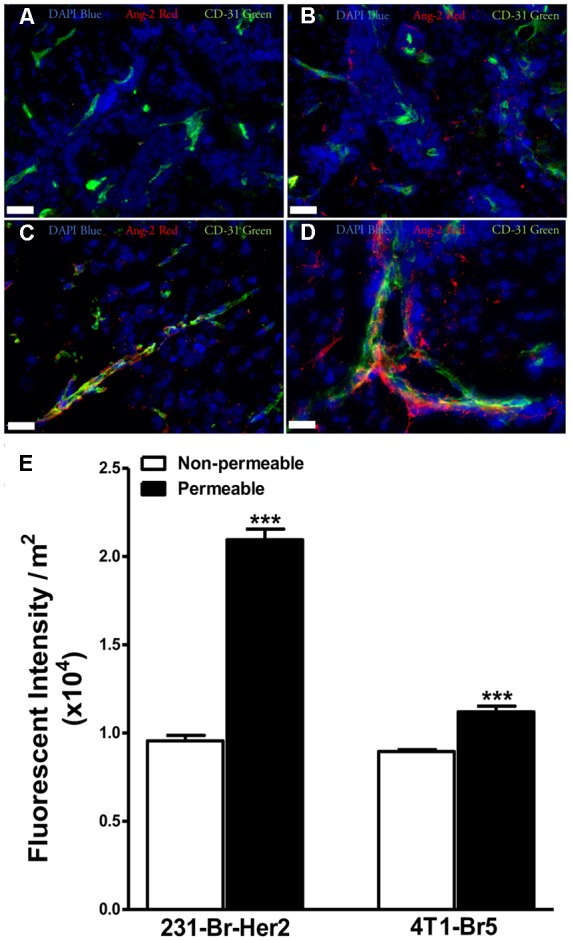
**Expression of angiopoietin-2 is greater in lesions with higher permeability.** Brain slices from un-treated metastases bearing animals were stained for the hypoxia induced vascular destabilizing protein Ang-2. Ang-2 expression in MDA-MB-231-BR-Her2 metastases is shown in non-permeable **(A)** and permeable **(B)** metastatic lesions. Representative images in the 4T1-BR5 model are shown in a lesion with low permeability **(C)** and a lesion with high permeability **(D)**. Ang-2 (red), CD31 (green), DAPI (blue). Ang-2 intensity in permeable and non-permeable lesion in both preclinical models of brain metastases of breast cancer **(E)**. Permeable lesions demonstrated greater Ang-2 expression than non-permeable in both models, with a >2-fold increase in the MDA-MB-231-BR-Her2 model, indicating the presence of vascular destabilization. Scale bar = 25 μm. Statistical significance was determined using Student’s *t*-test to determine difference in Ang-2 expression between the two permeability groups within each model (^∗∗∗^ for *p* < 0.001).

Because increases in VEGF expression have been reported to correlate with elevated blood-tumor barrier permeability of metastatic lesions, we wanted to determine the relative expression of Ang-2 in an experimental brain metastases of breast cancer model. Brain slices were stained with anti-Ang-2 and anti-CD31 antibodies. In addition, metastatic lesions were categorized either “non-permeable” or “permeable” based upon whether changes in ^14^C-AIB permeability exceeded the mean permeability of ^14^C-AIB in normal brain plus three standard deviations. Ang-2 staining was higher in permeable lesions than non-permeable in both models (**Figures 2A–E**). The presence of Ang-2 within metastatic lesions with higher permeability agrees with previous studies reporting the role of Ang-2 in vascular destabilization (Zhang et al., 2003).

### *In vivo* Inhibition of VEGF and Ang-2 Decreases ^14^C-AIB Permeability and Drug Uptake

**FIGURE 3:**
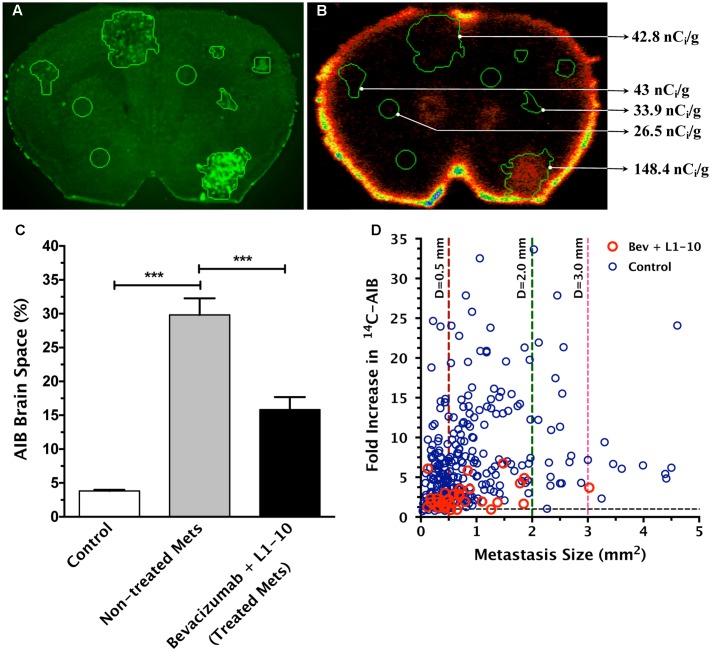
**Treatment of bevacizumab and L1-10AIB during metastasis development decreases permeability of ^14^C-AIB, which correlates with metastasis size.** Representative brain slice image of enhanced green fluorescent protein (eGFP) labeled lesions **(A)** and corresponding quantitative autoradiography (QAR) image **(B)** with ^14^C-AIB distribution. The percent brain space of ^14^C-AIB in normal brain and metastases **(C)**. Fold-increase in permeability with metastasis size reveals a weak correlation in bevacizumab plus L1-10 treated lesions (*n* = 65, *r*^2^ = 0.162, *p* < 0.05) **(D)**. Statistical significance was determined using ANOVA followed by Bonferroni’s multiple comparisons test for AIB brain space percentage, and linear regression analysis for fold increase in permeability versus lesion size (^∗∗∗^ for *p* < 0.001).

To determine the effects of VEGF and Ang-2 inhibition on blood-tumor barrier permeability *in vivo*, mice were injected with 1.75 × 10^5^ brain seeking cells followed by treatment of bevacizumab in combination with L1-10 10 days after inoculation. Representative images of eGFP labeled metastatic lesions in a treated brain are shown in **Figure 3A** and a representative autoradiograph of ^14^C-AIB permeability is shown in **Figure 3B**. In the treatment groups ^14^C-AIB permeability was significantly reduced to only ∼fourfold above control brain (**Figure 3C**). The highest observed fold change in the treatment group was 6.74 (**Figure 3D**) (compared to 33.7 in non-treated metastases).

**FIGURE 4:**
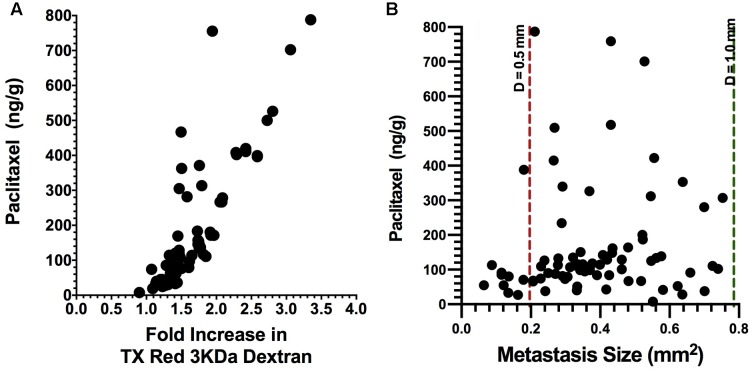
**Brain uptake of ^14^C-paclitaxel in brain metastases after bevacizumab treatment.** Bevacizumab reduces (*p* < 0.05) metastasis paclitaxel uptake to a mean of 163 ng/g, ranging from 7.76 to 788 ng/g (*n* = 81) as compared to our previously reported value of 276 ng/g ranging from 2.56 to 1768 (Lockman et al., 2010). In addition, there was a strong correlation with Texas Red 3 kDa dextran permeability (*r*^2^ = 0.72, *p* < 0.001) **(A)**. In addition, paclitaxel concentrations in metastases from the bevacizumab group showed a weak correlation with lesion size (*r*^2^ = 0.071, *p* < 0.05) **(B)**.

To determine if combinatorial therapy would similarly decrease brain uptake of paclitaxel, we repeated the studies of administering bevacizumab and L1-10 during metastasis development. On day 32, animals were administered ^14^C-paclitaxel 2 h prior to euthanasia and processed as described. ^14^C-Paclitaxel concentrations were significantly decreased by combination therapy of bevacizumab and L1-10 and standalone therapy of bevacizumab to a mean concentration of 202 and 163 ng/g, respectively (**Figure 4**) compared to untreated animals (276 ng/g, *P* < 0.05) (Lockman et al., 2010). Some lesions had uptake of more than 514 ng/g while others showed very little uptake 47 ng/g (compared with a range of 14.9 ng/g to 1961 ng/g in un-treated metastases).

**FIGURE 5:**
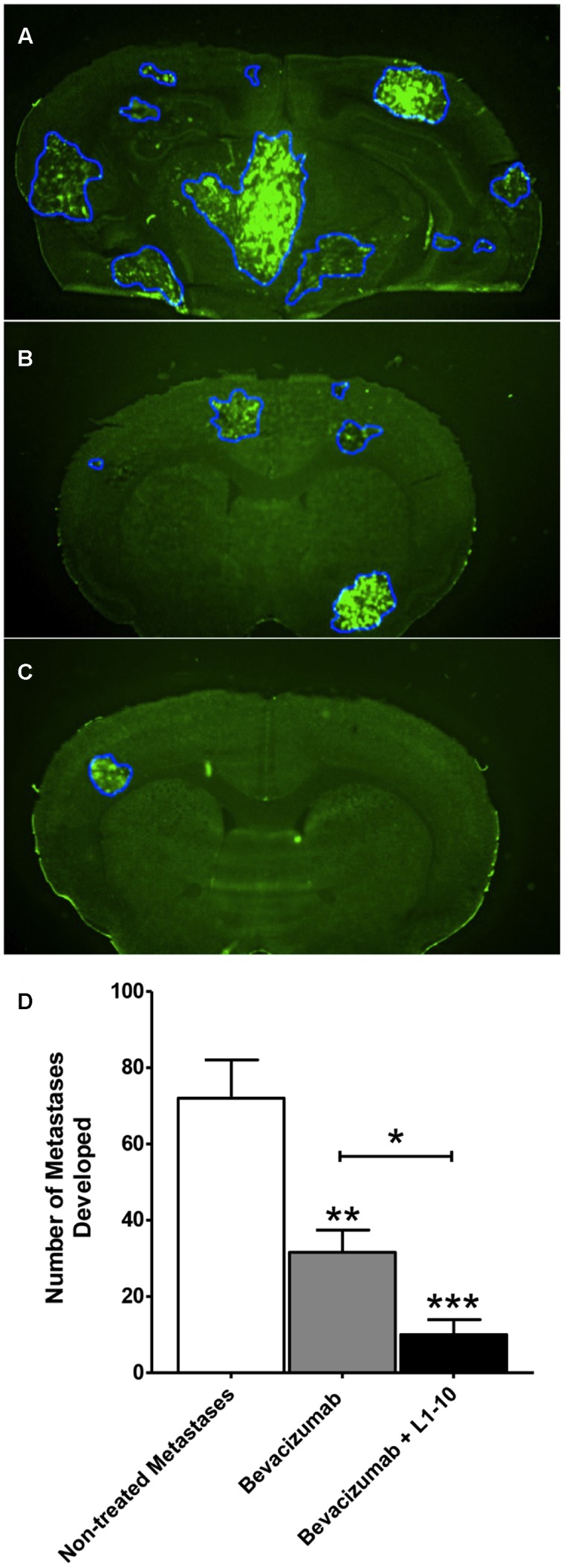
**Bevacizumab and L1-10 treatment reduces metastatic burden in brain. Representative images of MDA-MB-231-BR-Her2 non-treated metastatic brain** **(A)**, bevacizumab treated brain **(B)**, and bevacizumab + L1-10 treated brain **(C)**. Bevacizumab (10 mg/kg) or bevacizumab + L1-10 (4 mg/kg) was administered on day 10 after inoculation (with cancer cells and the number of metastatic lesions developed was significantly (*p* < 0.001) reduced in the bevacizumab alone group (56%) and in the combination group (86%) **(D)**. Treatment also reduced lesion size with 67% ± 1 of bevacizumab treated lesions and 78% ± 10 of bevacizumab + L1-10 treated lesions developing area < 0.5 mm^2^. Data illustrate a significant reduction in metastatic burden with simultaneous inhibition of the VEGF/angiopoietin-2 pathway. Significance was determined using ANOVA analysis followed by Bonferroni’s multiple comparisons test; ^∗^ for *p* < 0.05, ^∗∗^ for *p* < 0.01 and ^∗∗∗^ for *p* < 0.001.)

Importantly, fewer metastatic lesions developed (*p* < 0.001) in the combinatorial bevacizumab and L1-10 group (10 ± 4) than what was observed for either bevacizumab treatment (31.6 ± 6) or untreated controls (72 ± 10) (**Figure 5**). Furthermore, the percentage of large metastatic lesions present in the combinatorial group (7.7 ± 5) decreased (*p* < 0.05) relative to the percentage of large lesions observed in the bevacizumab treated group (14.6 ± 4) and significantly less (*p* < 0.05) than un-treated controls (34.6 ± 8).

## Discussion

In this study, we demonstrate that Ang-2 and VEGF work cooperatively to induce angiogenesis. We also show experimental brain metastases have activated Ang-2, that Ang-2 is present at the BTB vasculature, and blockade of the VEGF/Ang-2 pathway results in significant reductions in permeability, drug uptake, and metastatic burden.

Herein, we demonstrate that VEGF and Ang-2 have a combinational effect on angiogenesis, similar to previous reports (Chae et al., 2010; Hashizume et al., 2010; Morrissey et al., 2010). Additionally, we demonstrate that there was a greater inhibition of vessel branching in the presence of both bevacizumab and L1-10, indicating a potential role of L1-10 affecting several aspects of angiogenesis including tubule formation, endothelial cell proliferation, and migration of endothelial cells in monolayer. This is consistent with data from other reports demonstrating VEGF and Ang-2 can act to induce the formation of vascular tubes (Asahara et al., 1998; Lobov et al., 2002).

Hypoxia plays a role in several stages of tumor progression such as angiogenesis and metastatic invasion (Lu et al., 2010). Clinically, several cancer types such as lung, colon, and breast have been shown to contain regions of hypoxia compared to their adequately perfused counterparts (Zhong et al., 1999). Areas of hypoxia were also noted in bone metastases of breast cancer (Hiraga et al., 2007) and in animal models of gliomas (Bernsen et al., 2000). Data in our second set of experiments looking at hypoxia in preclinical brain metastases of breast cancer, agree with the clinical and other preclinical data that nearly every lesion has some degree of hypoxia whether or not it is adequately perfused (Brown, 1990; Hoückel and Vaupel, 2001; van Laarhoven et al., 2006; Lu et al., 2010).

Hypoxia has been shown to up-regulate Ang-2 mRNA in endothelial cells (Jones, 2003) as well as in rat brain tissue (Mandriota et al., 2000). Herein, we observed the presence of Ang-2 in the vasculature was associated with nearly all lesions identified in both preclinical models. Of interest, we observed elevated expression of Ang-2 in lesions with greater vascular permeability. Ang-2 up-regulation results from the induction of cyclooxygenase-2 (COX-2) enzyme, which metabolizes arachidonic acid to prostaglandin E2 in brain endothelial cells (LaManna et al., 2004). Clinically, aggressive gliomas have been shown to express greater levels of Ang-2 than low-grade gliomas; and *in vitro* studies suggest Ang-2 mRNA expression increases in human glioma cells in response to hypoxia (Koga et al., 2001). Under healthy conditions, Ang-2 is barely detectable in brain (Tait and Jones, 2004); however, VEGF has been shown to induce Ang-2 expression in brian (Mandriota and Pepper, 1998), resulting in both destabilization of brain vasculature and altering the integrity of the endothelium.

In this study, we demonstrate that lesions that have greater permeability in the 4T1BR model have increased Ang-2 expression. This data are consistent with literature demonstrating that during angiogenesis, Ang-2 is released from tumor cells (Morrissey et al., 2010) and binds the endothelial cell Tie2 receptor, which causes pericytes to pull away from the endothelium, resulting in a destabilized vasculature (Ahmad et al., 2001; Zhang et al., 2003; LaManna et al., 2004; Scharpfenecker et al., 2005). Vascular destabilization is further compounded when there is a decreased oxygen supply, which induces the dimerization of HIF-1α to HIF-1β, to form the HIF-1 complex. This dimer then translocates to the nucleus and acts as a transcription factor for VEGF and matrix metalloproteinases, which help to degrade the basement membrane (Zagzag et al., 2000; Kaur et al., 2005).

In our next set of experiments, we attempted to demonstrate that inhibition of VEGF with bevacizumab and Ang-2 with L1-10 results in decreased permeability and drug uptake. We observed that the combinatorial therapy resulted in a marked decrease in vascular permeability to ^14^C-AIB (∼100 Da) from ∼30-fold for lesions in untreated controls to ∼5-fold in the treatment group. We then evaluated whether the combinatorial therapy would also reduce paclitaxel uptake in the metastatic lesions. As expected, we saw a decreased uptake of paclitaxel within all lesions of combinatorial treatment groups (mean concentration of 202 ng/g, ranging from 47.1 to 514 ng/g) compared to the untreated controls (mean concentration of 392 ng/g, ranging from 14.9 to 1961 ng/g). Previous studies using identical models identified paclitaxel concentrations in brain metastases must exceed 1000 ng/g to induce apoptosis in the metastases (Lockman et al., 2010). In the current study, paclitaxel concentrations in both preclinical models failed to reach effective concentrations.

Previous reports have demonstrated that inhibition of VEGF results in slower tumor progression (Zhang et al., 2002; Holloway et al., 2006; Roland et al., 2009) and reduced metastasis development (Ellis et al., 2000; Verheul et al., 2007). Similarly, Ang-2 inhibition has been shown to slow prostate cancer (Morrissey et al., 2010) and glioma progression (Villeneuve et al., 2008). Therefore, we set out to determine whether inhibition of VEGF and Ang-2 by dual administration of bevacizumab plus L1-10, respectively, would reduce metastasis colonization and progression. We observed a significantly smaller number of lesions in the combinatorial therapy group (*n* = 10 ± 4) compared to the bevacizumab treated group (*n* = 31.6 ± 6) and compared to the untreated controls (*n* = 72 ± 10). These data suggest that simultaneous inhibition of VEGF and Ang-2 may be effective for the prevention of brain metastases.

In summary, we demonstrate an integral role of Ang-2 and VEGF in angiogenesis and brain metastases progression. While simultaneous inhibition of these two angiogenic factors may have significant potential in reducing the formation of experimental brain metastases, this strategy should not be used in established metastases since it significantly restricts permeability and drug uptake. Further work should be done to determine if simultaneous inhibition of the VEGF/Ang-2 pathway can be effectively employed to reduce brain metastasis formation altogether.

## Author Contributions

All authors listed, have made substantial, direct and intellectual contribution to the work, and approved it for publication.

## Conflict of Interest Statement

The authors declare that the research was conducted in the absence of any commercial or financial relationships that could be construed as a potential conflict of interest.
